# Medullary carcinomas of the nonampullary small intestine: association with coeliac disease, mismatch repair deficiency, PD‐L1 expression, and favourable prognosis

**DOI:** 10.1111/his.15307

**Published:** 2024-08-28

**Authors:** Alessandro Vanoli, Federica Grillo, Giuseppe De Lisi, Camilla Guerini, Giovanni Arpa, Catherine Klersy, Matteo Fassan, Paola Parente, Luca Mastracci, Elena Biletta, Gabriella Nesi, Maria C Macciomei, Marco V Lenti, Erica Quaquarini, Anna M Chiaravalli, Daniela Furlan, Stefano La Rosa, Marco Paulli, Antonio Di Sabatino

**Affiliations:** ^1^ Department of Molecular Medicine University of Pavia Pavia Italy; ^2^ Unit of Anatomic Pathology IRCCS San Matteo Hospital Foundation Pavia Italy; ^3^ IRCCS Ospedale Policlinico San Martino Genoa Italy; ^4^ Pathology Unit, Department of Surgical Sciences and Integrated Diagnostics (DISC) University of Genoa Genoa Italy; ^5^ Anatomic Pathology Unit of Pavia Institute Istituti Clinici Scientifici Maugeri IRCCS Pavia Italy; ^6^ Biostatistics and Clinical Trial Center Fondazione IRCCS Policlinico San Matteo Pavia Italy; ^7^ Department of Medicine (DIMED), Surgical Pathology & Cytopathology Unit University of Padua Padua Italy; ^8^ Veneto Institute of Oncology IOV‐IRCCS Padua Italy; ^9^ Unit of Pathology Fondazione IRCCS Ospedale Casa Sollievo della Sofferenza San Giovanni Rotondo Italy; ^10^ Unit of Pathology, Department of Surgery ASL BI Nuovo Ospedale degli Infermi Ponderano Italy; ^11^ Pathology Section, Department of Health Sciences University of Florence Florence Italy; ^12^ Pathology Unit San Camillo‐Forlanini Hospital Rome Italy; ^13^ Department of Internal Medicine and Medical Therapeutics University of Pavia Pavia Italy; ^14^ Department of Internal Medicine IRCCS San Matteo Hospital Foundation Pavia Italy; ^15^ Medical Oncology Unit Istituti Clinici Scientifici Maugeri IRCCS Pavia Italy; ^16^ Department of Oncology Ospedale di Circolo, ASST‐Sette Laghi Varese Italy; ^17^ Hereditary Cancer Research Center University of Insubria Varese Italy; ^18^ Unit of Pathology, Department of Medicine and Technological Innovation University of Insubria Varese Italy

**Keywords:** ARID1A, Epstein–Barr virus, immune‐mediated disorders, microsatellite instability, small bowel adenocarcinoma

## Abstract

**Aim:**

Gastrointestinal medullary carcinoma is a rare histologic subtype of adenocarcinoma. As nonampullary small bowel medullary carcinomas (SB‐MCs) are poorly characterized, we aimed to analyse their clinicopathologic and immunohistochemical features and to compare them with nonmedullary small bowel adenocarcinomas (NM‐SBAs).

**Methods and Results:**

Surgically resected SBAs collected through the Small Bowel Cancer Italian Consortium were classified as SB‐MCs (carcinomas with ≥50% of tumour fulfilling the typical histologic criteria of MC) or NM‐SBAs. Immunohistochemistry for cytokeratin (CK)7, CK20, CDX2, programmed death‐ligand 1 (PD‐L1) and mismatch repair proteins was performed in both SB‐MCs and NM‐SBAs. SB‐MCs were also tested for CK8/18, synaptophysin, SMARCB1, SMARCA2, SMARCA4, and ARID1A and for Epstein–Barr virus (EBV)‐encoded RNAs by *in‐situ* hybridization. *MLH1* promoter methylation status was evaluated in MLH1‐deficient cases. Eleven SB‐MCs and 149 NM‐SBAs were identified. One (9%) SB‐MC was EBV‐positive, while 10 (91%) harboured mismatch repair deficiency (dMMR). *MLH1* promoter hypermethylation was found in all eight dMMR SB‐MCs tested. Switch/sucrose nonfermentable deficiency was seen in two (18%) SB‐MCs, both with isolated loss of ARID1A. Compared with NM‐SBAs, SB‐MCs exhibited an association with coeliac disease (*P* < 0.001), higher rates of dMMR (*P* < 0.001), and PD‐L1 positivity by both tumour proportion score and combined positive score (*P* < 0.001 for both), and a lower rate of CK20 expression (*P* = 0.024). Survival analysis revealed a better prognosis of SB‐MC patients compared to NM‐SBA cases (*P* = 0.02).

**Conclusion:**

SB‐MCs represent a distinct histologic subtype, with peculiar features compared to NM‐SBAs, including association with coeliac disease, dMMR, PD‐L1 expression, and better prognosis.

## Graphical abstract

Nonampullary small bowel medullary carcinomas are associated with coeliac disease and show higher rates of mismatch repair deficiency and PD‐L1 positivity, as well as a more favourable outcome, compared to nonmedullary cases. They should be considered a distinct subtype of small bowel adenocarcinoma.

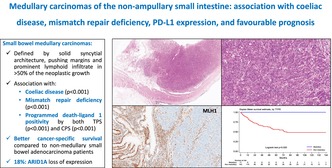

**Abbreviations**
CK: cytokeratin
CoeD: coeliac disease
CPS: combined positive score
dMMR: mismatch repair deficiency
EBER: EBV‐encoded small RNAs
EBV: Epstein‐Barr virus
MC: medullary carcinoma
MMR: mismatch repair
MSI: microsatellite instability
NM‐SBA: nonmedullary small bowel adenocarcinoma
PD‐L1: programmed death‐ligand 1
pMMR: mismatch repair proficient
SBA: small bowel adenocarcinoma
SBA‐NOS: small bowel adenocarcinoma
SB‐MC: small bowel medullary carcinoma
SWI/SNF: switch/sucrose nonfermentable
TIL: tumor‐infiltrating lymphocyte
TPS: tumour proportion score
WHO: World Health Organization

## Introduction

In the 5th edition of the World Health Organization (WHO) classification of Digestive System Tumours, the term “medullary carcinoma” (MC) indicates an uncommon, distinct histologic subtype of adenocarcinoma, which may be found in the large bowel, in the stomach, and, more rarely, in the small intestine or the pancreas.^1^ The typical histologic features of gastrointestinal MC (so‐called for the low‐power microscopic resemblance to “*medulla oblongata*”), are the following: (i) a solid or nested tumour architecture; (ii) a pushing‐type (circumscribed) tumour border; (iii) a cellular stroma rich in tumour‐infiltrating lymphocytes (TILs) and sometimes other inflammatory cells; and (iv) relatively uniform tumour cells with a syncytial appearance, vesicular nuclei, prominent nucleoli, and relatively abundant eosinophilic cytoplasm.^2^, ^3^

Colorectal MCs appear to be underdiagnosed,^4^ likely due to a lack of familiarity with morphologic diagnostic criteria and difficulties in distinguishing them from poorly differentiated conventional adenocarcinomas.^2^, ^5^ Nevertheless, they have been consistently associated with deficient mismatch repair (dMMR) status, higher Programmed‐Death‐Ligand1 (PD‐L1) expression,^6^, ^7^, ^8^ and a better prognosis.^9^, ^10^, ^11^ In the stomach, cancers with MC‐type histology are currently referred to as “(adeno)carcinomas with lymphoid stroma”, which are strongly associated with Epstein–Barr virus (EBV) positivity or dMMR, as well as with high PD‐L1 expression.^1^ Moreover, in the colon, switch/sucrose nonfermentable (SWI/SNF)‐deficiency (i.e. the loss of expression of the SWI/SNF complex subunits, a family of chromatin remodellers including BRG‐1/BRM‐associated factor complex subunits and ARID1A) has been shown to be also associated with medullary or undifferentiated/rhabdoid phenotype, dMMR, and a worse prognosis.^12^, ^13^, ^14^, ^15^, ^16^

While clinicopathologic features of ampullary MCs have been thoroughly described by Xue *et al*.,^17^ nonampullary small bowel MCs (SB‐MCs) are still poorly known, with very few case reports or small series,^18^, ^19^, ^20^, ^21^, ^22^, ^23^, ^24^, ^25^ suggesting an association with dMMR, PD‐L1 expression, and improved outcome, while the potential relevance of SWI/SNF‐deficiency in SB‐MCs has not yet been investigated.

The present study aimed to investigate the clinicopathologic, immunophenotypic and molecular features of a relatively large series of nonampullary SB‐MCs, and to compare them with those of nonampullary nonmedullary small bowel adenocarcinomas (NM‐SBAs).

## Materials and Methods

### Study population

This retrospective multicentre study enrolled 160 patients (part of whom had already entered previous studies), who underwent surgical resection for primary, nonampullary SBA from Centres participating in the Small Bowel Cancer Italian Consortium. Neuroendocrine neoplasms, adenosquamous/squamous, and undifferentiated carcinomas were excluded from the study, which was approved by the Ethics Committee of Pavia (protocol number: 20140003980). Written informed consent was obtained.

### Histologic subtype definition

A central histopathological review of all tumours was performed by three pathologists (A.V., G.A., and S.L.R.) and two pathology residents (G.D.L. and C.G.), and any discordance in evaluation was solved by consensus.

An SBA was classified as SB‐MC if at least 50% of the tumour showed all the typical features of MCs described in the rest of the gastrointestinal tract (i.e. (i) a solid or nested tumour architecture; (ii) a pushing‐type tumour border; (iii) a TIL‐enriched stroma; and (iv) relatively uniform tumour cells with a syncytial appearance, with vesicular nuclei and prominent nucleoli), as proposed by Lee *et al*. for colorectal medullary carcinomas and by Xue *et al*. for ampullary medullary carcinomas.^2^, ^17^ SB‐MCs were also assessed for the presence of glandular, mucinous, or signet‐ring cell components, tumour necrosis, eosinophilic or neutrophilic infiltrates (distant from tumour necrosis), as well as for adjacent dysplastic lesions. The remaining NM‐SBAs were classified as WHO histologic subtypes, and SBAs, not otherwise specified (SBAs‐NOS) were subdivided into low‐grade (≥50% gland formation) and high‐grade (<50% gland formation) tumours.^1^, ^26^, ^27^

### Clinicopathologic data

For all cases, clinical‐demographic data, including patient gender and age at SBA diagnosis, aetiologic factors, and tumour site, lymphovascular, and perineural invasion, American Joint Committee on Cancer (8th edition) pTNM stage, as well as survival data, were obtained from pathologic reports and follow‐up clinical reports.

### Immunohistochemistry

Four‐μm‐thick tissue whole sections of all cancers (with available tumour sections) were immunostained on a Dako Omnis platform (Agilent, Santa Clara, CA, USA) with the following antibodies: cytokeratin (CK)7 (clone: OV‐TL‐12/30, prediluted, Dako), CK20 (Js20.8, prediluted, Dako), CDX2 (DAK‐CDX2, prediluted, Dako), MLH1 (ES05, prediluted, Dako), MSH2 (FE11, prediluted, Dako), MSH6 (EP49, prediluted, Dako), PMS2 (364 EP51, prediluted, Dako), and PD‐L1 (22C3, prediluted, Dako). In addition, SB‐MCs were tested for CK8/18 (EP17/EP30, prediluted, Dako), synaptophysin (DAK‐SYNAP, prediluted, Dako), and the SWI/SNF components SMARCB1/INI1 (EPR12014‐77, 1:1000, Abcam, Cambridge, UK), SMARCA2/BMR (polyclonal, 1:400, Sigma, St. Louis, MO, USA), SMARCA4/BRG1 (EPNCIR111A, 1:200, Abcam), and ARID1A (polyclonal, 1:400, Sigma).

The extent of staining for CK7, CK20, and CDX2 was at first scored semiquantitatively (no staining; 1–10%; 11–50%; >50%); only carcinomas with >10% of tumour cells showing expression were considered positive, as previously reported.^28^ Immunostaining for MMR proteins was considered MMR‐proficient (pMMR) if unequivocal nuclear expression of all four MMR proteins was retained, or dMMR if complete loss of nuclear expression of one or more MMR proteins was observed, in the presence of an adequate internal positive control. PD‐L1 expression was scored as tumour proportion score (TPS) and combined positive score (CPS).^29^ An SBA was considered PD‐L1 positive by TPS if the TPS was ≥1% and by CPS if the CPS was ≥1. For the interpretation of immunoexpression of SWI/SNF components, only unequivocal absent staining in the viable tumour nuclei, in the presence of a strong nuclear staining of stromal/inflammatory cells, was considered “deficient”.^15^

### EBV *in situ* hybridization, microsatellite instability (MSI), and *MLH1* promoter methylation

SB‐MCs were analysed for EBV‐encoded small RNAs (EBERs) by *in situ* hybridization, for MSI by polymerase chain reaction and, for those showing loss of expression of MLH1, for *MLH1* promoter methylation status, as previously described.^30^, ^31^

### Statistical analysis

All analyses were performed using the Stata software (release 18, StataCorp, College Station, TX, USA). A 2‐sided *P*‐value was considered statistically significant. For multiple comparisons, the Bonferroni correction applied.

Continuous variables were reported as median and 25th–75th percentiles (25th–75th) and compared between SB‐MCs and NM‐SBA groups with the Mann–Whitney *U*‐test. Categorical variables were reported as counts and percent and compared with the Fisher exact test. Survival curves were plotted using the Kaplan–Meier method and compared between groups with the log‐rank test. Given the null mortality in SB‐MCs, no hazard ratios (Cox model) could be computed.

## Results

### Clinicopathologic, immunophenotypic, and molecular characteristics of SB‐MCs

**Figure 1:**
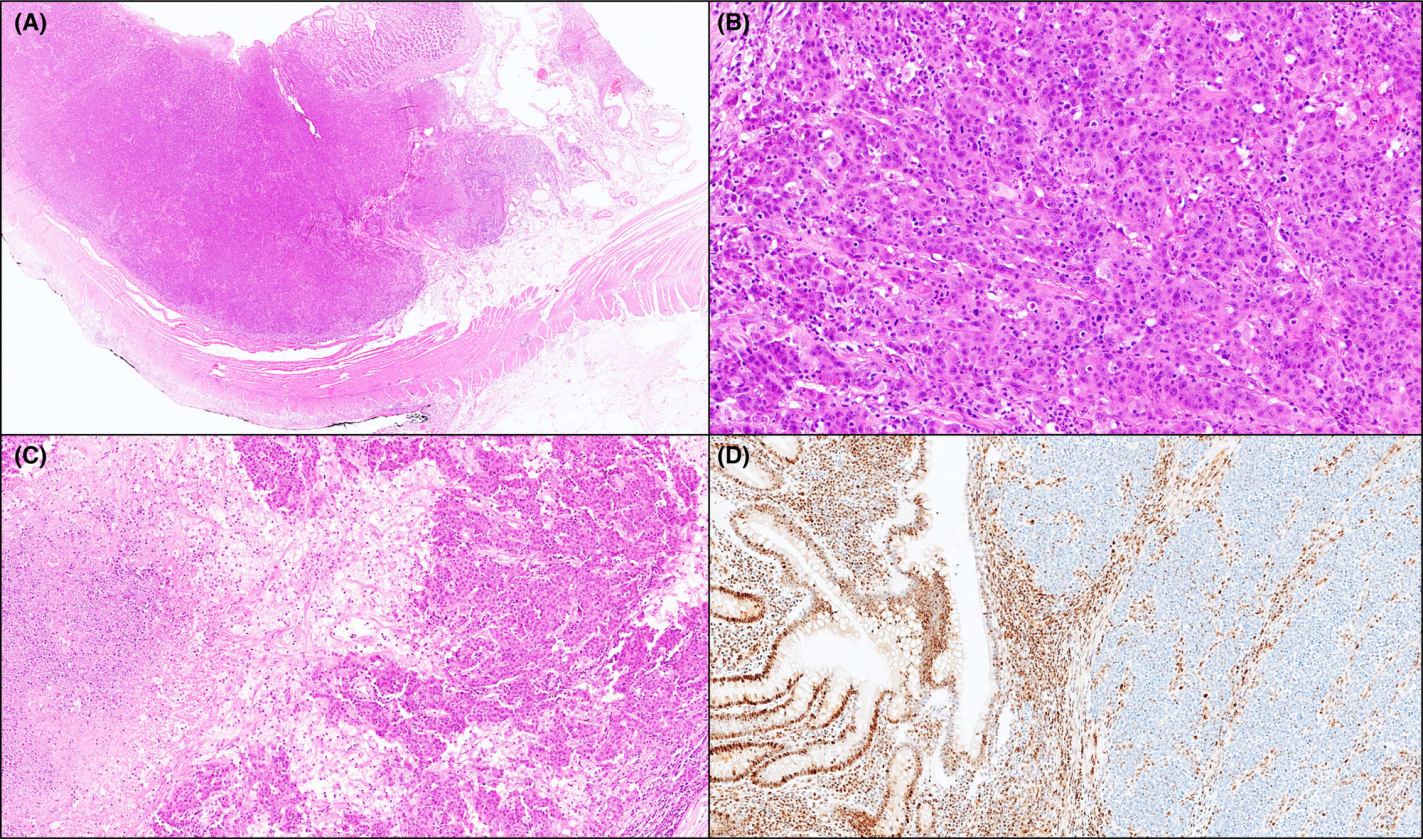
A duodenal medullary carcinoma in a coeliac patient. A pushing‐type tumour border is seen (A, hematoxylin and eosin [H&E]). The neoplasm features a solid structure with syncytial appearance of tumour cells, admixed with a nondesmoplastic stroma enriched in inflammatory cells, including lymphocytes and neutrophils (B, H&E). Areas of tumour necrosis are present (C, H&E). Tumour cells show loss of expression of MLH1, while MLH1 is retained in the adjacent mucosa and the inflammatory infiltrate (internal control) (D, MLH1 immunostaining). [Color figure can be viewed at wileyonlinelibrary.com]

**Figure 2:**
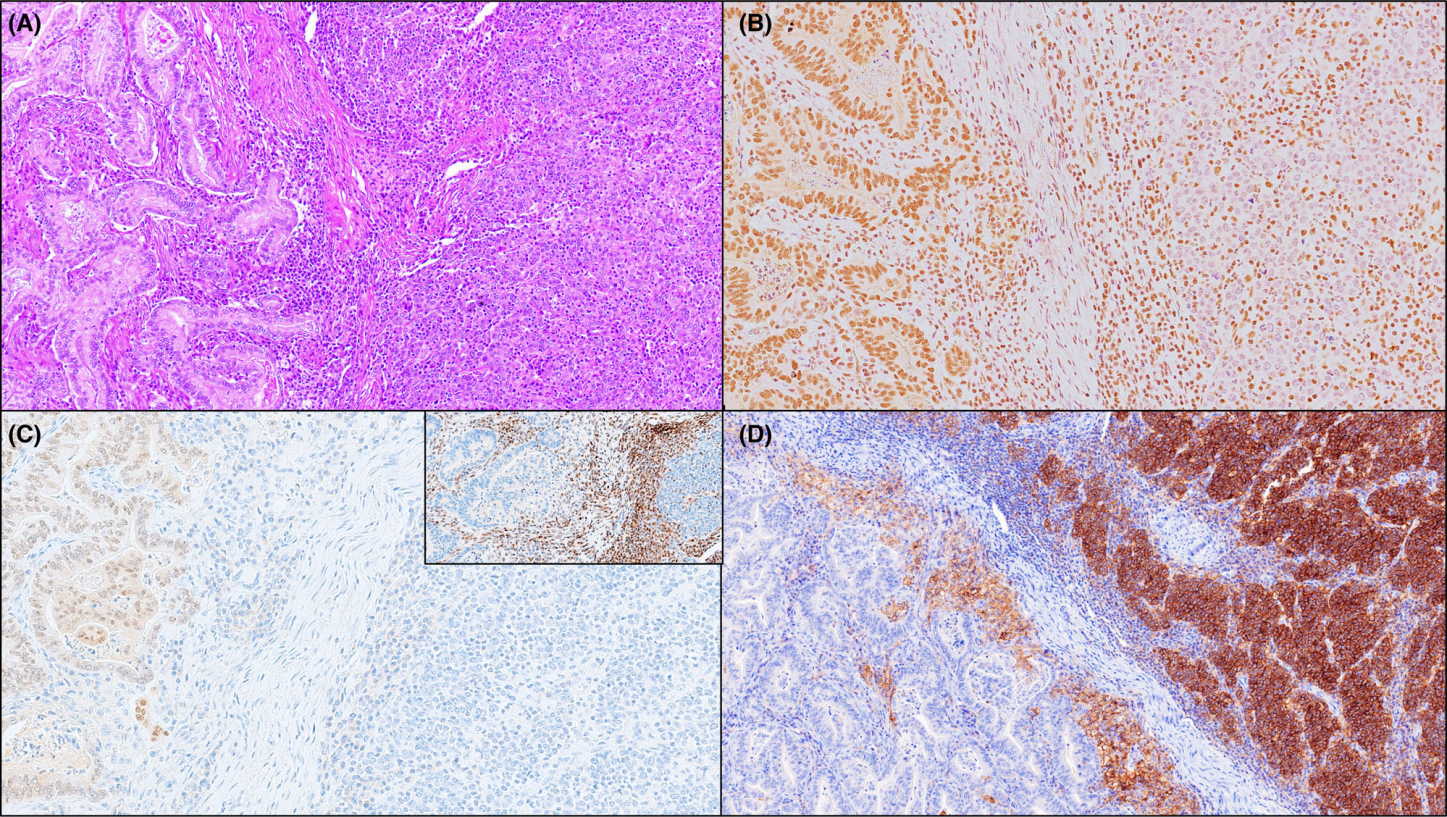
A jejunal medullary carcinoma associated with coeliac disease, with a glandular component. The glandular area (on the left; A, H&E) is focal, accounting for 10% of tumour surface, and exhibits retained ARID1A (B, ARID1A immunostaining) and CDX2 (C, CDX2 immunostaining) expression, whereas the medullary component shows lack of both proteins. Both components feature loss of MLH1 expression (inset of C; MLH1 immunostaining). Strong PD‐L1 membranous expression by neoplastic cells was only observed in the medullary component (D, PD‐L1 immunostaining). [Color figure can be viewed at wileyonlinelibrary.com]

Among the 160 SBAs, 11 (7%) SB‐MCs were found. The clinicopathologic features of MCs are outlined in Table 1. The median patient age at SB‐MC diagnosis was 66 years, with a slight prevalence of females (55%). Most of them arose in the jejunum (64%) and in coeliac disease (CoeD) patients (73%). Histologically, medullary features accounting for ≥90% of the tumour burden were observed in 10/11 (91%) SB‐MCs, while in the remaining case medullary features accounted for 70%. Apart from the characteristics typical of medullary histology (Figure 1), four cases showed glandular components (10% of tumour surface in three cases and 30% in one case) (Figure 2), whereas mucinous and signet‐ring differentiation was not seen. The dysplastic component adjacent to the carcinoma was either absent (nine cases) or limited (two cases). Tumour necrosis (Figure 1C) and neutrophilic infiltrates were a very frequent finding (detected in 8 out of 11 cases), while prominent eosinophilic infiltrate was more rarely observed (in 2 out of 11 cases). In one case, the tumour stroma was essentially composed of T lymphocytes and scattered plasma cells. SB‐MCs were diagnosed in pT3 (nine cases, 82%) or pT4 (two cases, 18%) stage, with locoregional lymph node metastases in 45% of cases.

**Table 1:** Comparison of clinicopathologic and immunohistochemical features between medullary and nonmedullary small bowel adenocarcinomas

	SB‐MCs (*n* = 11)	Nonmedullary SBAs (*n* = 149)	*P*‐value*	dMMR SB‐MCs (*n* = 10)	*P*‐value^†^
Patient age at SBA diagnosis, median (25th–75th)	66 (52–80)	62 (52–72)	0.438	66.5 (52–80)	0.440
Female gender, *N* (%)	6/11 (55%)	54/149 (36%)	0.333	6/10 (60%)	0.179
Tumour site, *N* (%)					
Duodenum	2/11 (20%)	18/149 (12%)	0.133	2 (20%)	**0.046**
Jejunum	7/11 (64%)	61/149 (41%)		7 (70%)	
Ileum	2/11 (20%)	70/149 (47%)		1 (10%)	
Aetiology, *N* (%)					
Crohn's disease	2/11 (18%)	52/149 (35%)	**0.006**	1/10 (10%)	**0.002**
Coeliac disease	8/11 (73%)	32/149 (21%)		8/10 (80%)	
Lynch syndrome	0/11 (0%)	15/149 (10%)		0/10 (0%)	
No predisposing condition	1/11 (9%)	50/149 (34%)		1/10 (10%)	
Lymph node metastases, *N* (%)	5/11 (45%)	67/149 (45%)	1.000	4/10 (40%)	1.000
AJCC stage, *N* (%)					
I	0/11 (0%)	11/149 (7%)	0.447	0/10 (0%)	0.492
II	6/11 (55%)	69/149 (47%)		6/10 (60%)	
III	5/11 (45%)	51/149 (34%)		4/10 (40%)	
IV	0/11 (0%)	18/149 (12%)		0/10 (0%)	
Lymphovascular invasion, *N* (%)	4/11 (36%)	104/149 (70%)	**0.04**	3/10 (30%)	**0.015**
Perineural invasion, *N* (%)	2/11 (18%)	57/149 (38%)	0.331	2/10 (20%)	0.325
CDX2 expression, *N* (%)	5/11 (45%)	104/148 (70%)	0.101	4/10 (40%)	0.074
CK20 expression, *N* (%)	3/11 (27%)	94/148 (63%)	**0.024**	3/10 (30%)	**0.046**
CK7 expression, *N* (%)	1/10 (10%)	48/138 (35%)	0.166	1/9 (11%)	0.273
dMMR, *N* (%)	10/11 (91%)	47/149 (31%)	**<0.001**	NA	NA
PD‐L1 TPS ≥ 1%	7/11 (64%)	4/113 (3%)	**<0.001**	6/10 (60%)	**<0.001**
PD‐L1 CPS ≥ 1	9/11 (82%)	25/113 (22%)	**<0.001**	8/10 (80%)	**<0.001**

Bold values indicate statistical significance. AJCC, American Joint Committee on Cancer; CK, Cytokeratin; CPS, Combined positive score; dMMR, Mismatch repair deficiency; NA, Not applicable; SBA, Small bowel adenocarcinoma; SB‐MCs, Medullary carcinomas; TPS, Tumour proportion score.

* SB‐MCs versus nonmedullary SBAs.

^†^ dMMR SB‐MCs versus nonmedullary SBAs.

**Figure 3:**
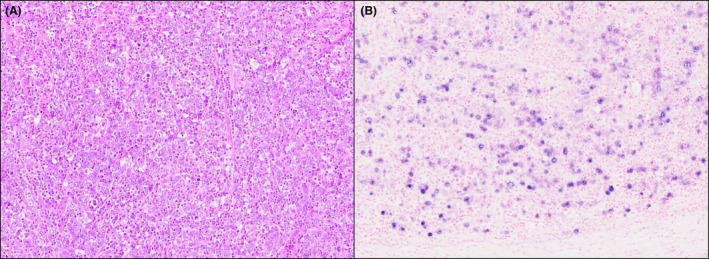
An ileal carcinoma in a patient with Crohn's disease, showing the typical histologic features of medullary carcinoma. Note the relative uniformity of tumour cells with a syncytial appearance, vesicular nuclei, prominent nucleoli, as well as a conspicuous cellular stroma rich in inflammatory cells (A, H&E). Tumour cells are positive for EBER (B, EBER *in‐situ* hybridization). [Color figure can be viewed at wileyonlinelibrary.com]

Ten (91%) SB‐MCs were classified as dMMR, as they showed a combined loss of MLH1 and PMS2 expression by all cancer cells (Figure 1D). In all dMMR SB‐MCs with available tumour sections for molecular analyses (eight cases), both MSI and *MLH1* promoter hypermethylation were found. A single pMMR SB‐MC (arising in a patient with Crohn's disease) proved to be EBER‐positive (Figure 3), while all the other MCs were EBER‐negative. All SB‐MCs expressed CK8/18, while no case showed synaptophysin positivity. Expression of all SWI/SNF complex proteins was intact in nine SB‐MCs, whereas two cases (both dMMR CoeD‐associated SB‐MCs) exhibited isolated ARID1A loss. Of note, one of two MCs with ARID1A loss revealed retained ARID1A expression in its focal glandular component, while both components exhibited loss of MLH1 expression (Figure 2).

### Comparisons between SB‐MCs and NM‐SBAs

The comparison between SB‐MCs and NM‐SBAs (Table 1) revealed a significant association between medullary histology and aetiology (*P* = 0.006), with a significantly higher prevalence of SB‐MCs among CoeD patients (8 out of 40 cases, 20%) compared to nonCoeD cases (3 out of 120 cases, 2.5%, *P* < 0.001). Lymphovascular invasion (*P* = 0.04) and CK20 expression (*P* = 0.024) were significantly less frequently identified in SB‐MCs. In addition, SB‐MCs featured significantly higher rates of dMMR (*P* < 0.001) and PD‐L1 positivity by TPS (*P* < 0.001) or CPS (*P* < 0.001) compared to NM‐SBAs. These differences between SB‐MCs and NM‐SBAs remained significant even after exclusion of the EBV‐positive carcinoma from the SB‐MC group (Table 1). Worthy of note, three (27%) MCs had a TPS >10% (Figure 2D) and two of them (18%), including the EBV‐positive MC, exhibited a TPS of 90%.

Interestingly, when compared to high‐grade SBAs‐NOS only (*n* = 34), SB‐MCs showed significantly lower rates of lymphovascular invasion (26% versus 91%, *P* = 0.001), CK20 expression (27% versus 66%, *P* = 0.038), and significantly higher rates of dMMR (91% versus 34%, *P* = 0.002) and PD‐L1 positivity by TPS (64% versus 4%, *P* < 0.001) and CPS (82% versus 22%, *P* = 0.002). When comparisons were restricted to dMMR cases (*n* = 47), dMMR SB‐MCs (*n* = 10) were characterized by significantly higher rates of PD‐L1 positivity by TPS and CPS and significantly less common CDX2 expression compared to nonmedullary cases (Table 2). Finally, compared to CoeD‐associated NM‐SBAs (*n* = 32), CoeD‐associated SB‐MCs (*n* = 8) exhibited significantly higher rates of dMMR (100% versus 53%, *P* = 0.016), PD‐L1 positivity by TPS (62% versus 3%, *P* = 0.001) and by CPS (87% versus 28%, *P* = 0.004), and significantly less frequent CDX2 expression (50% versus 87%, *P* = 0.037).

**Table 2:** Comparison of clinicopathologic and immunohistochemical features between medullary and nonmedullary mismatch repair‐deficient small bowel adenocarcinomas

	dMMR SB‐MCs (*n* = 10)	dMMR nonmedullary SBAs (*n* = 47)	*P*‐value
Patient age at SBA diagnosis, median (25th–75th)	66.5 (52–80)	58 (52–75)	0.514
Female gender, *N* (%)	6/10 (60%)	19/47 (40%)	0.308
Tumour site, *N* (%)			
Duodenum	2 (20%)	7 (15%)	0.198
Jejunum	7 (70%)	22 (47%)	
Ileum	1 (10%)	18 (38%)	
Aetiology, *N* (%)			
Crohn's disease	1/10 (10%)	9/47 (19%)	0.053
Coeliac disease	8/10 (80%)	17/47 (36%)	
Lynch syndrome	0/10 (0%)	15/47 (32%)	
No predisposing condition	1/10 (10%)	6/47 (13%)	
Lymph node metastases, *N* (%)	4/10 (40%)	15/47 (32%)	0.717
AJCC stage, *N* (%)			
I	0/10 (0%)	6/47 (13%)	0.464
II	6/10 (60%)	26/47 (55%)	
III	4/10 (40%)	12/47 (26%)	
IV	0/10 (0%)	3/47 (6%)	
Lymphovascular invasion, *N* (%)	3/10 (30%)	26/47 (55%)	0.179
Perineural invasion, *N* (%)	2/10 (20%)	11/47 (23%)	1.000
CDX2 expression, *N* (%)	4/10 (40%)	38 (81%)	**0.015**
CK20 expression, *N* (%)	3/10 (30%)	23 (49%)	0.319
CK7 expression, *N* (%)	1/9 (11%)	8/37 (22%)	0.664
PD‐L1 TPS ≥ 1%	6/10 (60%)	3/31 (10%)	**0.003**
PD‐L1 CPS ≥ 1	8/10 (80%)	10/31 (32%)	**0.012**

Bold values indicate statistical significance. AJCC, American Joint Committee on Cancer; CK, Cytokeratin; CPS, Combined positive score; dMMR, Mismatch repair deficiency; SBA, Small bowel adenocarcinoma; SB‐MCs, Small bowel medullary carcinomas; TPS, Tumour proportion score.

### Survival analysis

**Figure 4:**
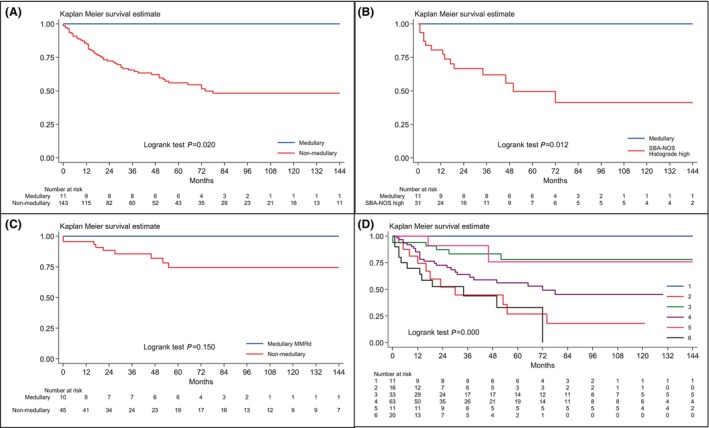
Kaplan–Meier cancer‐specific survival estimates. (A) A comparison between SB‐MC patients and NM‐SBA patients. (B) A comparison between SB‐MC patients and patients with high‐grade SBA‐NOS. (C) A comparison between dMMR SB‐MC patients and dMMR NM‐SBA patients. (D) Comparison of six SBA subgroups based on histotype, histologic grade, and MMR status. 1: SB‐MCs; 2: poorly cohesive carcinomas; 3: Low‐grade dMMR SBAs‐NOS; 4: Low‐grade pMMR SBAs‐NOS; 5: High‐grade dMMR SBAs‐NOS; 6: High‐grade pMMR SBAs‐NOS. [Color figure can be viewed at wileyonlinelibrary.com]

Patients were followed up for a median time of 62 months (25th–75th: 27–116 months). No MC patient died of cancer. Cancer‐specific survival proved to be significantly more favourable for MC patients in comparison with NM‐SBA (*P* = 0.020, Figure 4A) or high‐grade SBA‐NOS patients (*P* = 0.012, Figure 4B). These survival differences were confirmed also after the exclusion of the EBV‐positive SB‐MC patient. However, no significant survival difference was found between dMMR MCs and dMMR NM‐SBAs (*P* = 0.150, Figure 4C) or between CoeD‐associated MCs and CoeD‐associated NM‐SBAs (*P* = 0.341). When SBAs were subdivided into six subgroups based on histotype, histologic grade, and MMR status, significant survival differences emerged (*P* < 0.001, Figure 4D). *Post‐hoc* comparisons showed a more favourable survival of MC compared to poorly cohesive carcinomas (*P* = 0.001) and to high‐grade pMMR SBA‐NOS cases (*P* = 0.001), whereas no significant difference was seen between MC and either low‐grade or high‐grade dMMR SBA‐NOS patients (*P* = 0.153 and *P* = 0.178, respectively). Finally, a better outcome of MC patients compared to low‐grade pMMR SBA‐NOS cases was seen (*P* = 0.017), which, however, lost significance when Bonferroni's correction was applied.

## Discussion

In the present study, based on a relatively large series of SB‐MCs, we found several distinctive features of SB‐MCs compared to the remaining SBAs.

First, in the small intestine a clinically relevant association between medullary histology and CoeD, a known predisposing condition for SBA development,^32^ emerged, strengthening suggestions from our previous reports.^19^, ^22^ In our study, a relevant fraction (20%) of CoeD‐associated SBAs (8 out of 40 cases collected in numerous Italian CoeD Centres) were MCs. On the contrary, among SBAs with no predisposing conditions and Crohn's disease‐associated SBAs, medullary histology seems to be much less common (2% and 4% of cases, respectively), and no SB‐MC associated with Lynch syndrome was identified.

Second, SB‐MCs appear to follow two distinct and mutually exclusive pathogenetic pathways, similar to those of the stomach and ampullary region.^17^, ^33^ The first pathway, which accounts for the vast majority (91%) of SB‐MCs and for all CoeD‐associated cases, is the dMMR/MSI pathway, essentially related to epigenetic inactivation of the *MLH1* gene. The dMMR/MSI phenotype proved to be significantly more frequent among SB‐MCs, even when the analysis was restricted to CoeD‐associated SBAs, which are known to be enriched in dMMR.^31^, ^34^ The other pathway, involving a much smaller number of SBAs with medullary‐type histology (9%), is driven by EBV infection. In the literature, only a very few case reports of such EBV‐positive SBAs, often labelled as “lymphoepithelioma‐like carcinomas” due to their similarity with EBV‐positive nasopharyngeal carcinoma, are available.^35^, ^36^, ^37^ In the present investigation, we decided to include among SB‐MCs the single EBV‐positive carcinoma (fulfilling all MC criteria and previously reported^30^, ^35^), due to the well‐known difficulties in distinguishing, “*a priori*”, i.e. on morphologic grounds only, the two different carcinogenic pathways. Indeed, in the stomach the term “carcinoma with lymphoid stroma” has been adopted by the current WHO classification to encompass both MSI‐related (“medullary”) and EBV‐related (“lymphoepithelioma‐like”) poorly differentiated cancers characterized by prominent lymphocytic infiltrate.^1^

Importantly, our findings show that SB‐MCs behave as low‐grade tumours, even though they appear poorly differentiated, with frequent tumour necrosis, likely as a consequence of their high TIL density. Indeed, despite the lack of a significant difference in tumour stage, MC patients displayed a significantly better outcome compared to NM‐SBA, high‐grade SBA‐NOS, and poorly cohesive carcinoma cases, as well as showing a trend towards a more favourable prognosis compared to low‐grade pMMR SBA‐NOS patients. No significant survival difference, however, was seen between SB‐MCs and either low‐grade or high‐grade dMMR SBAs‐NOS.

Another relevant finding of the present investigation was the significantly higher rate of PD‐L1 positivity by both TPS and CPS in SB‐MCs compared to NM‐SBAs, confirming, on a larger series, previous findings from our group,^38^ and others.^21^ These differences also persisted when comparisons were restricted to dMMR or to CoeD‐associated cases only, indicating a distinctive, immunoregulatory microenvironment of gastrointestinal MCs,^39^ with potential therapeutic implications.

SB‐MCs must be distinguished from small bowel undifferentiated/rhabdoid carcinomas, most of which are SWI/SNF‐deficient and have a more ominous prognosis.^15^, ^40^ The relevance of SWI/SNF‐deficiency in SB‐MCs seems to be more limited, with isolated ARID1A loss involving 18% of our SB‐MCs. Kim *et al*. found ARID1A loss in a similar fraction (20%) of NM‐SBAs and that it was associated with poor prognosis and signet ring cell and undifferentiated carcinomas,^41^ while Gonzalez *et al*. reported ARID1A loss in 7% of all SBAs and in a single case (8%) out of 12 SBAs with medullary differentiation.^42^ In one of our SB‐MCs (Figure 2), ARID1A expression was absent in the medullary component, while it is retained in the focal glandular component, suggesting that the two morphologically distinct components may be associated with different carcinogenetic pathways. More comprehensive studies on SWI/SNF‐deficiency in SBAs are needed, as this molecular phenotype is emerging as a promising predictive biomarker for immuno‐oncologic therapy.^43^

Finally, similar to ampullary MCs,^17^ nonampullary SB‐MCs rarely have recognizable preinvasive components, suggesting that they might not follow the classical adenoma‐adenocarcinoma sequence, and they are frequently negative for CDX2 and CK20, which must be borne in mind in such cases so as not to misinterpret them as extraintestinal in origin. In addition, the high percentage of negativity for CDX2 of small/large bowel MCs and undifferentiated/rhabdoid carcinomas may aid in their differential diagnosis with poorly differentiated conventional adenocarcinomas, most of which are CDX2‐positive.^15^, ^44^

Our study has several limitations, including its retrospective nature and the relatively small number of SB‐MCs; however, to the best of our knowledge, this is the largest series of these very rare tumours hitherto described. Another limitation could be related to the potentially low diagnostic reproducibility in distinguishing SB‐MCs from high‐grade SBAs‐NOS, similar to colorectal MCs.^2^, ^5^, ^45^ Nevertheless, to mitigate this issue we performed a centralized histologic review of all cases, resulting in a consensus diagnosis.

In conclusion, SB‐MC, despite its rarity, is worthy of being considered as a distinct subtype of nonampullary SBA by virtue of its association with CoeD, its high prevalence of *MLH1* methylation‐driven dMMR and PD‐L1 expression, and its favourable outcome.

## Author contributions

Concept and design of the study: AV. Acquisition of data, or analysis and interpretation of data: all authors. Drafting the article: AV, FG, GDL, CG, SLR, ADS, MP. Statistical analysis: CC. Figure preparation: AV, GDL, EQ. Revising the article critically for important intellectual content: all authors. Final review and approval of the version submitted: all authors.

## Funding information

This study was supported by grants from the Italian Ministry of Health through “Fondazione IRCCS Policlinico San Matteo, Pavia, Italy” to A.V. and to A.D.S. This study was also partly supported by a grant of the Italian Ministry of Education, University and Research (MIUR) to the Department of Molecular Medicine of the University of Pavia under the initiative “Dipartimenti di Eccellenza (2018–2022).”

## Conflict of interest

The authors disclose that they have no significant relationships with, or financial interest in, any commercial companies pertaining to this article.

## Data Availability

The datasets used and/or analysed during the current study are available from the corresponding author upon reasonable request.

## References

[1] WHO Classification of Tumours Editorial Board . Digestive system tumours. WHO classification of tumours series. Vol. 1. 5th ed. Lyon, France: International Agency for Research on Cancer, 2019.

[2] Lee LH , Yantiss RK , Sadot E *et al*. Diagnosing colorectal medullary carcinoma: interobserver variability and clinicopathological implications. Hum. Pathol. 2017; 62; 74–82. 28034727 10.1016/j.humpath.2016.12.013 PMC5392420

[3] Chetty R . Gastrointestinal cancers accompanied by a dense lymphoid component: an overview with special reference to gastric and colonic medullary and lymphoepithelioma‐like carcinomas. J. Clin. Pathol. 2012; 65; 1062–1065. 22918886 10.1136/jclinpath-2012-201067

[4] Scott N , West NP , Cairns A , Rotimi O . Is medullary carcinoma of the colon underdiagnosed? An audit of poorly differentiated colorectal carcinomas in a large national health service teaching hospital. Histopathology 2021; 78; 963–969. 33247957 10.1111/his.14310

[5] Fiehn AM , Grauslund M , Glenthøj A , Melchior LC , Vainer B , Willemoe GL . Medullary carcinoma of the colon: can the undifferentiated be differentiated? Virchows Arch. 2015; 466; 13–20. 25339302 10.1007/s00428-014-1675-6

[6] Rosenbaum MW , Bledsoe JR , Morales‐Oyarvide V , Huynh TG , Mino‐Kenudson M . PD‐L1 expression in colorectal cancer is associated with microsatellite instability, BRAF mutation, medullary morphology and cytotoxic tumor‐infiltrating lymphocytes. Mod. Pathol. 2016; 29; 1104–1112. 27198569 10.1038/modpathol.2016.95

[7] Lee LH , Cavalcanti MS , Segal NH *et al*. Patterns and prognostic relevance of PD‐1 and PD‐L1 expression in colorectal carcinoma. Mod. Pathol. 2016; 29; 1433–1442. 27443512 10.1038/modpathol.2016.139 PMC5083129

[8] Roberts J , Salaria SN , Cates J *et al*. PD‐L1 expression patterns in microsatellite instability‐high intestinal adenocarcinoma subtypes. Am. J. Clin. Pathol. 2019; 152; 384–391. 31152546 10.1093/ajcp/aqz052 PMC6669412

[9] Lanza G , Gafà R , Matteuzzi M , Santini A . Medullary‐type poorly differentiated adenocarcinoma of the large bowel: a distinct clinicopathologic entity characterized by microsatellite instability and improved survival. J. Clin. Oncol. 1999; 17; 2429–2438. 10561306 10.1200/JCO.1999.17.8.2429

[10] Knox RD , Luey N , Sioson L *et al*. Medullary colorectal carcinoma revisited: a clinical and pathological study of 102 cases. Ann. Surg. Oncol. 2015; 22; 2988–2996. 25572685 10.1245/s10434-014-4355-5

[11] Pyo JS , Sohn JH , Kang G . Medullary carcinoma in the colorectum: a systematic review and meta‐analysis. Hum. Pathol. 2016; 53; 91–96. 27001432 10.1016/j.humpath.2016.02.018

[12] Ahadi MS , Fuchs TL , Clarkson A *et al*. Switch/sucrose‐nonfermentable (SWI/SNF) complex (SMARCA4, SMARCA2, INI1/SMARCB1)‐deficient colorectal carcinomas are strongly associated with microsatellite instability: an incidence study in 4508 colorectal carcinomas. Histopathology 2022; 80; 906–921. 34951482 10.1111/his.14612

[13] Agaimy A , Sheahan K . SWI/SNF deficiency: potential new biomarker in a subset of mismatch repair‐deficient colorectal carcinomas. Histopathology 2022; 80; 905. 35434884 10.1111/his.14649

[14] Chou A , Toon CW , Clarkson A *et al*. Loss of ARID1A expression in colorectal carcinoma is strongly associated with mismatch repair deficiency. Hum. Pathol. 2014; 45; 1697–1703. 24925223 10.1016/j.humpath.2014.04.009

[15] Agaimy A , Daum O , Märkl B , Lichtmannegger I , Michal M , Hartmann A . SWI/SNF complex‐deficient undifferentiated/rhabdoid carcinomas of the gastrointestinal tract: a series of 13 cases highlighting mutually exclusive loss of SMARCA4 and SMARCA2 and frequent co‐inactivation of SMARCB1 and SMARCA2. Am. J. Surg. Pathol. 2016; 40; 544–553. 26551623 10.1097/PAS.0000000000000554

[16] Villatoro TM , Ma C , Pai RK . Switch/sucrose nonfermenting nucleosome complex‐deficient colorectal carcinomas have distinct clinicopathologic features. Hum. Pathol. 2020; 99; 53–61. 32222462 10.1016/j.humpath.2020.03.009

[17] Xue Y , Balci S , Pehlivanoglu B *et al*. Medullary carcinoma of the ampulla has distinct clinicopathologic characteristics including common association with microsatellite instability and PD‐L1 expression. Hum. Pathol. 2023; 131; 38–46. 36502926 10.1016/j.humpath.2022.12.004

[18] Peycru T , Jarry J , Soubeyran I . Sporadic medullary carcinoma of the ileum. Clin. Gastroenterol. Hepatol. 2011; 9; A24. 10.1016/j.cgh.2011.03.006 21397730

[19] Brcic I , Cathomas G , Vanoli A , Jilek K , Giuffrida P , Langner C . Medullary carcinoma of the small bowel. Histopathology 2016; 69; 136–140. 26599717 10.1111/his.12908

[20] Xue Y , Vanoli A , Balci S *et al*. Non‐ampullary‐duodenal carcinomas: clinicopathologic analysis of 47 cases and comparison with ampullary and pancreatic adenocarcinomas. Mod. Pathol. 2017; 30; 255–266. 27739441 10.1038/modpathol.2016.174

[21] Thota R , Gonzalez RS , Berlin J , Cardin DB , Shi C . Could the PD‐1 pathway be a potential target for treating small intestinal adenocarcinoma? Am. J. Clin. Pathol. 2017; 148; 208–214. 28821192 10.1093/AJCP/AQX070

[22] Vanoli A , Di Sabatino A , Martino M *et al*. Small bowel carcinomas in celiac or Crohn's disease: distinctive histophenotypic, molecular and histogenetic patterns. Mod. Pathol. 2017; 30; 1453–1466. 28664941 10.1038/modpathol.2017.40

[23] Jun SY , Park ES , Lee JJ *et al*. Prognostic significance of stromal and intraepithelial tumor‐infiltrating lymphocytes in small intestinal adenocarcinoma. Am. J. Clin. Pathol. 2020; 153; 105–118. 31576398 10.1093/ajcp/aqz136

[24] Colarossi C , Mare M , La Greca G *et al*. Medullary carcinoma of the gastrointestinal tract: report on two cases with immunohistochemical and molecular features. Diagnostics (Basel) 2021; 11; 1775. 34679473 10.3390/diagnostics11101775 PMC8534691

[25] Liu L , Kaur S , Dayyani F *et al*. Medullary carcinoma of the duodenum treated with pembrolizumab: a case report. J. Gastrointest. Oncol. 2023; 14; 1149–1154. 37201040 10.21037/jgo-22-755 PMC10186505

[26] College of American Pathologists . Protocol for the examination of specimens from patients with carcinoma of the small intestine. (Accessed February 7, 2024). Available at: https://documents.cap.org/protocols/Small_Int_4.2.0.0.REL_CAPCP.

[27] Vanoli A , Guerini C , Grillo F *et al*. Poorly cohesive carcinoma of the nonampullary small intestine: a distinct histologic subtype with prognostic significance. Am. J. Surg. Pathol. 2022; 46; 498–508. 34628432 10.1097/PAS.0000000000001821

[28] Overman MJ , Pozadzides J , Kopetz S *et al*. Immunophenotype and molecular characterisation of adenocarcinoma of the small intestine. Br. J. Cancer 2010; 102; 144–150. 19935793 10.1038/sj.bjc.6605449 PMC2813754

[29] Mastracci L , Grillo F , Parente P *et al*. PD‐L1 evaluation in the gastrointestinal tract: from biological rationale to its clinical application. Pathologica 2022; 114; 352–364. 36305021 10.32074/1591-951X-803 PMC9614301

[30] Vanoli A , Di Sabatino A , Martino M *et al*. Epstein‐Barr virus‐positive ileal carcinomas associated with Crohn's disease. Virchows Arch. 2017; 471; 549–552. 28752215 10.1007/s00428-017-2209-9

[31] Vanoli A , Di Sabatino A , Furlan D *et al*. Small bowel carcinomas in coeliac or Crohn's disease: clinico‐pathological, molecular, and prognostic features. A study from the Small Bowel Cancer Italian Consortium. J. Crohns Colitis 2017; 11; 942–953. 28333239 10.1093/ecco-jcc/jjx031

[32] Emilsson L , Semrad C , Lebwohl B , Green PHR , Ludvigsson JF . Risk of small bowel adenocarcinoma, adenomas, and carcinoids in a nationwide cohort of individuals with celiac disease. Gastroenterology 2020; 159; 1686–1694.e2. 32679218 10.1053/j.gastro.2020.07.007

[33] Hissong E , Ramrattan G , Zhang P *et al*. Gastric carcinomas with lymphoid stroma: an evaluation of the histopathologic and molecular features. Am. J. Surg. Pathol. 2018; 42; 453–462. 29438172 10.1097/PAS.0000000000001018

[34] Potter DD , Murray JA , Donohue JH *et al*. The role of defective mismatch repair in small bowel adenocarcinoma in celiac disease. Cancer Res. 2004; 64; 7073–7077. 15466202 10.1158/0008-5472.CAN-04-1096

[35] Vanoli A , Di Sabatino A , Biancone L *et al*. Small bowel Epstein‐Barr virus‐positive lympho‐epithelioma‐like carcinoma in Crohn's disease. Histopathology 2017; 70; 837–839. 27891660 10.1111/his.13133

[36] Chaparro Mirete M , López‐López V , Robles Campos R . Lymphoepithelioma‐like carcinoma of the duodenum: a very infrequent tumor. Rev. Esp. Enferm. Dig. 2020; 112; 239. 32022569 10.17235/reed.2020.6503/2019

[37] Kwei‐Nsoro R , Wang Y , Gandhi S , Ojemolon P , Awoyomi M , Ogar A . Epstein‐Barr virus‐positive lymphoepithelioma‐like carcinoma in celiac disease. ACG Case Rep. J. 2023; 10; e00970. 36777463 10.14309/crj.0000000000000970 PMC9911188

[38] Giuffrida P , Arpa G , Grillo F *et al*. PD‐L1 in small bowel adenocarcinoma is associated with etiology and tumor‐infiltrating lymphocytes, in addition to microsatellite instability. Mod. Pathol. 2020; 33; 1398–1409. 32066859 10.1038/s41379-020-0497-0

[39] Friedman K , Brodsky AS , Lu S *et al*. Medullary carcinoma of the colon: a distinct morphology reveals a distinctive immunoregulatory microenvironment. Mod. Pathol. 2016; 29; 528–541. 26965581 10.1038/modpathol.2016.54

[40] Chang B , Sheng W , Wang L *et al*. SWI/SNF complex‐deficient undifferentiated carcinoma of the gastrointestinal tract: clinicopathologic study of 30 cases with an emphasis on variable morphology, immune features, and the prognostic significance of different SMARCA4 and SMARCA2 subunit deficiencies. Am. J. Surg. Pathol. 2022; 46; 889–906. 34812766 10.1097/PAS.0000000000001836

[41] Kim MJ , Gu MJ , Chang HK , Yu E . Loss of ARID1A expression is associated with poor prognosis in small intestinal carcinoma. Histopathology 2015; 66; 508–516. 25400081 10.1111/his.12566

[42] González I , Goyal B , Xia MD , Pai RK , Ma C . DNA mismatch repair deficiency but not ARID1A loss is associated with prognosis in small intestinal adenocarcinoma. Hum. Pathol. 2019; 85; 18–26. 30381262 10.1016/j.humpath.2018.10.013

[43] Agaimy A . SWI/SNF‐deficient malignancies: optimal candidates for immune‐oncological therapy? Adv. Anat. Pathol. 2023; 30; 211–217. 36069856 10.1097/PAP.0000000000000366

[44] Winn B , Tavares R , Fanion J *et al*. Differentiating the undifferentiated: immunohistochemical profile of medullary carcinoma of the colon with an emphasis on intestinal differentiation. Hum. Pathol. 2009; 40; 398–404. 18992917 10.1016/j.humpath.2008.08.014 PMC2657293

[45] Remo A , Fassan M , Vanoli A *et al*. Morphology and molecular features of rare colorectal carcinoma histotypes. Cancers (Basel) 2019; 11; 1036. 31340478 10.3390/cancers11071036 PMC6678907

